# Advantages of hybrid intravascular ultrasound-optical coherence tomography system in clinical practice

**DOI:** 10.3389/fcvm.2025.1595889

**Published:** 2025-07-04

**Authors:** Yangfeng Xie, Wenbo Han, Shuai Wang, Wenhao Jia, Yunxiao Wang, Jie Li, Buxing Chen

**Affiliations:** Department of Cardiology, Beijing University of Chinese Medicine Third Affiliated Hospital, Beijing, China

**Keywords:** IVUS-OCT, hybrid intracoronary imaging system, coronary atherosclerotic heart disease, plaque characteristics, immediate post-stent evaluation

## Abstract

**Background:**

While the hybrid intravascular ultrasound-optical coherence tomography (IVUS-OCT) imaging system offers theoretical advantages for enhanced characterization of vascular morphology and histopathology through multimodal integration, its clinical efficacy lacks systematic validation. We conducted a comprehensive comparative analysis of this novel hybrid imaging modality against conventional single-modality OCT and IVUS systems, aiming to establish an evidence-based foundation for its clinical implementation and broader adoption in interventional cardiology practice.

**Objective:**

To evaluate the clinical advantages of hybrid intravascular ultrasound-optical coherence tomography (IVUS-OCT) system compared with single-modality imaging techniques in clinical practice.

**Methods:**

The hybrid IVUS-OCT intracoronary imaging system was employed to evaluate the characteristics of coronary atherosclerotic plaques and the immediate post-stent outcomes and compared against single-modality OCT and IVUS. The post-stent immediate effects were evaluated by the clear stent capture rate (CSCR), identification of incomplete stent apposition, tissue protrusion, and stent edge dissection.

**Results:**

74 patients underwent successful hybrid imaging (82 vessels imaged). Plaque analysis (23 vessels) identified 41 plaques [21 lipid, 20 calcified, 2 thin-cap fibroatheromas (TCFAs)]. OCT alone detected 21 lipid, 16 calcified, 3 possible TCFAs (maximal calcified arc accuracy: 68.75%). IVUS alone detected 15 lipid, 20 calcified, 0 TCFAs (maximal calcified arc accuracy: 85%). For post-stent evaluation (74 vessels), hybrid imaging visualized all stents (CSCR = 100%), detecting 23 incomplete stent apposition, 10 tissue protrusions, and 10 edge dissections. OCT detected 66 CSCR (89.19%), 23 incomplete stent apposition (100%), 10 tissue protrusions (100%), and 10 edge dissections (100%). IVUS detected 37 CSCR (50%), 8 incomplete stent apposition (34.78%), 2 tissue protrusions (20%), and 7 edge dissections (70%). Hybrid IVUS-OCT and OCT significantly outperformed IVUS in CSCR, tissue protrusion, and incomplete stent apposition detection (*P* < 0.05).

**Conclusion:**

The hybrid IVUS-OCT intracoronary imaging system outperforms single-modality IVUS or OCT in evaluating coronary atherosclerotic plaque characteristics and immediate post-stent outcomes.

## Graphical Abstract

**Figure:**
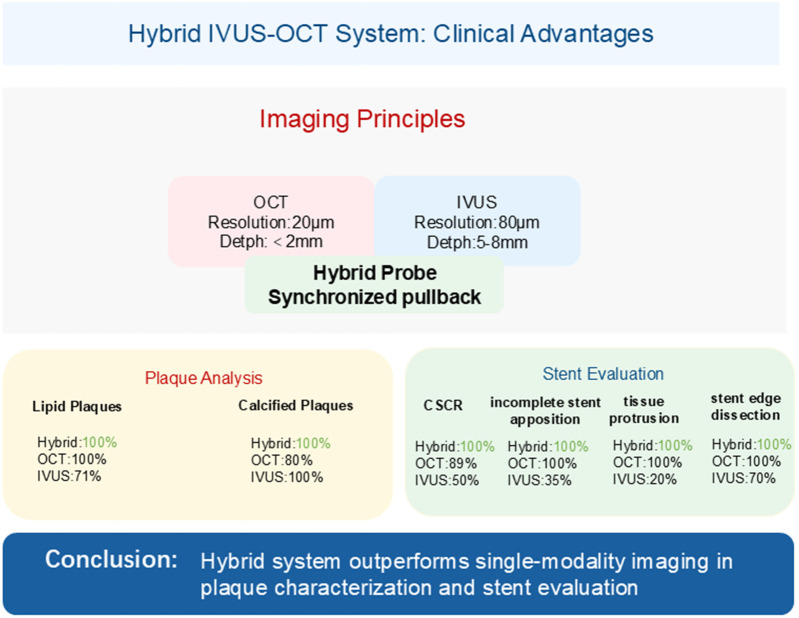
Hybrid IVUS-OCT Clinical Advantages

## 1 Introduction

Optical coherence tomography (1, 2) (OCT) and intravascular ultrasound (3) (IVUS) are increasingly used in clinical practice (4). OCT utilizes broadband near-infrared light (wavelengths typically in the vicinity of 1,310 nm) and low-coherence interferometry to detect tissue reflections, achieving high axial resolution (10–20 μm) for detailed visualization of vascular endothelium and plaque microstructures, though limited by light scattering to penetration depths <2 mm. Conversely, IVUS utilizes high-frequency ultrasound (typically 20–40 MHz) to penetrate deeper tissue (5–8 mm) by detecting acoustic impedance variations at tissue interfaces, but exhibits lower resolution (axial: 80–150 μm) (5) Multiple randomized controlled studies (6, 7) have confirmed that percutaneous coronary interventions (PCI) guided by IVUS or OCT optimization achieved better clinical outcomes. The updated 2024 European Society of Cardiology (ESC) Guidelines for the management of chronic coronary syndromes affirm the immediate and long-term benefits of IVI-guided PCI. They strongly recommend (Class I, Level A) the use of intravascular ultrasound (IVUS) or optical coherence tomography (OCT) for PCI in patients with complex coronary anatomy, particularly those with left main disease, true bifurcations, and long lesions (8).

However, each technology has its limitations; IVUS offers deeper penetration, enabling visualization of the entire vessel wall and detection of deep calcification, but is hampered by lower resolution, which impedes reliable differentiation between superficial and deep calcification (9). Conversely, OCT provides higher resolution (10), but its limited penetration depth may cause the outer margin of calcified plaques to be mistaken for lipid plaques, potentially leading to suboptimal selection of modification devices and under-expanded stents (11). Therefore, combining both techniques enables multimodal imaging that delivers more comprehensive histological and morphological information about vessels (12, 13). We evaluated the clinical application of the hybrid IVUS-OCT intracoronary imaging system by comparing with single mode of OCT and IVUS, aiming to provide a reference for clinical application and promotion.

## 2 Materials and methods

### 2.1 Study subjects

The data for this study were sourced from the Department of Cardiology, Third Affiliated Hospital of Beijing University of Chinese Medicine. Patients with borderline coronary lesions requiring further evaluation or severe coronary stenosis requiring percutaneous coronary intervention (PCI) were enrolled between October 2023 and December 2024. Exclusion criteria were: (1) Cardiogenic shock, severe hepatic or renal insufficiency; (2) Contrast agent allergy; (3) Difficulty in performing hybrid IVUS-OCT intravascular imaging (chronic total occlusion or extreme vessel tortuosity); (4) Poor image quality (e.g., inadequate blood clearance). Seventy-seven patients were initially enrolled. After excluding 3 patients with unclear OCT imaging, 74 patients were ultimately included in this study. This study was approved by the hospital ethics committee (approval number: BZYSY-2022KYKTPJ-09), conducted in accordance with the Declaration of Helsinki, and written informed consent was obtained from all participants.

### 2.2 Study equipment and procedure

**Figure 1:**
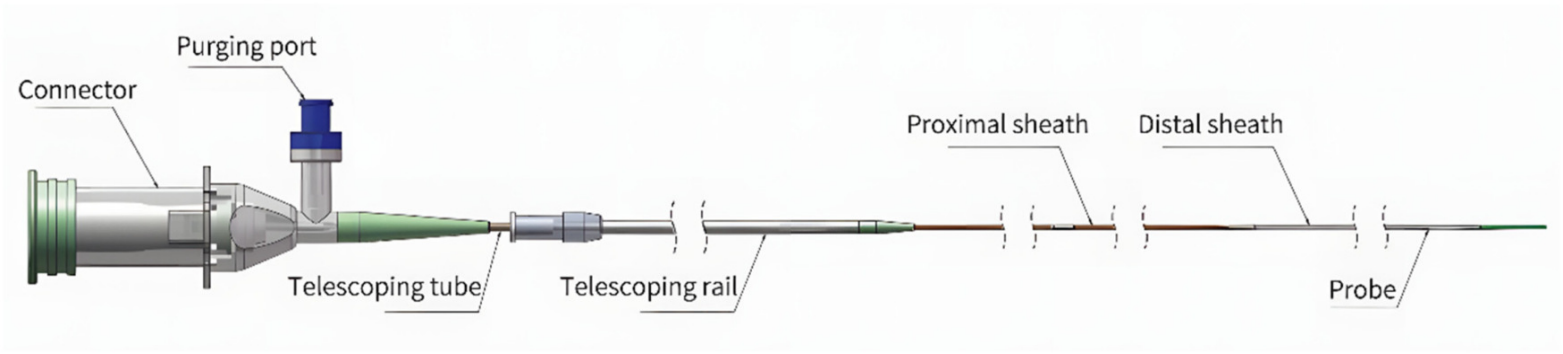
Catheter design and imaging characteristics frame rate: 200 fps; pullback speed: Up to 40 mm/s; pullback length: Up to 150 mm; IVUS axial resolution: 80 μm; OCT axial resolution: 20 μm.

Following routine angiography, hybrid IVUS-OCT intracoronary imaging was performed using the S1 system (Panovision Co., Ltd, Beijing, China) equipped with a C1-1 hybrid catheter (outer diameter: 0.98 mm). This catheter integrates sequentially arranged OCT and IVUS probes at its tip, enabling simultaneous acquisition of co-registered IVUS and OCT images during a single pullback. Synchronous imaging is achieved through two key mechanisms: (1) probe scanning angle alignment, achieved by precision catheter manufacturing ensuring identical directional orientation of both probes; and (2) pullback synchronization, wherein a two-frame offset is algorithmically compensated through software adjustments ( Figure 1 ).

The operator advanced the catheter to the distal target vessel, and contrast agent was injected to obtained clear images of the vessel on the S1 system. The procedure was performed at a retraction speed of 20 mm/s. Image data were analyzed offline by two independent researchers using Micro DICOM Viewer. If their opinions differed, a third expert was consulted for evaluation.

### 2.3 Image analysis

#### 2.3.1 Coronary plaque characteristics analysis

After completing the hybrid IVUS-OCT imaging, the minimum lumen diameter of the target vessel was identified, and the lesions were analyzed frame by frame with a 0.2 mm interval. Two calcifications were considered as parted when they were longitudinally separated by at least 1 mm or when they were detectable on different portions of the single slice image without any contact or continuity throughout the whole length of the calcifications themselves (14). Separate analyses were first conducted on the OCT and IVUS components to determine plaque characteristics independently for each modality. Subsequently, plaque features were characterized based on the coregistered IVUS-OCT images. These results were then compared. Based on the IVUS-OCT image results, the maximal calcified plaque arc location in the target vessel was determined, and the maximal calcified plaque arc measured by OCT imaging and IVUS imaging were compared (15). Finally, the presence of thin-cap fibroatheroma (TCFA) was analyzed.

In OCT images (16, 17), lipid plaques appeared as low-signal areas with blurry border, while calcified plaques were characterized by well-defined border with low-signal areas. In IVUS images (18), lipid plaques typically appear as low-echo plaques, and calcified plaques showed high-echo lesions with acoustic shadowing. TCFA is defined as a large lipid plaque with a fibrous cap thickness of less than 65 μm and macrophages.

#### 2.3.2 Immediate post-stent evaluation

The hybrid IVUS-OCT imaging system was also additionally employed for evaluating immediate post-stent outcomes, including clear stent capture rate (CSCR) (19), identification of incomplete stent apposition, tissue protrusion, and stent edge dissection. The hybrid IVUS-OCT cross-sectional images were analyzed separately to assess stent visualization, apposition, and edge dissection. Any tissue protrusion was also recorded. The results were compiled and analyzed to determine the effectiveness of the hybrid IVUS-OCT intracoronary imaging system in post-stent evaluation.

The CSCR refers to the ability to clearly visualize the entire stent in the target vessel. In OCT, this is indicated by a bright scattered light from the stent's inner wall, with reflective shadows behind the stent struts, while in IVUS, it is seen as a high-echo signal from the stent. Tissue protrusion (20, 21) occurs when vessel wall tissue protrudes into the lumen due to stent strut penetration, with irregular morphology and varying echo intensity. Incomplete stent apposition (22) is defined as a distance greater than the strut thickness between the stent struts and the vessel wall, with a distance ≥200 μm, and blood flow behind the stent struts (excluding flow over the side branches). Stent edge dissection (23) refers to a discontinuity in the vessel lumen surface at the stent edge (including a 5 mm segment at both the proximal and distal edges of the stent), leading to intimal tears or subintimal hematomas.

### 2.4 Statistical methods

Data analysis was performed using SPSS 26.0 software. Categorical data were expressed as frequencies and percentages, while continuous data were presented as mean ± standard deviation. Chi-square tests or Fisher's tests were used for categorical data analysis, with statistical significance set at *P* < 0.05.

## 3 Results

### 3.1 Basic information and imaging results

**Figure 2:**
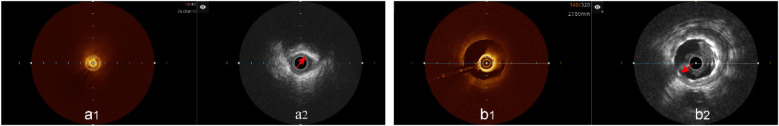
Unclear OCT imaging; IVUS shows plaque characteristics and stents. **(a1)** Unclear OCT imaging; **(a2)** IVUS shows calcified plaque; **(b1)** Unclear OCT imaging; **(b2)** IVUS shows stent.

A total of 77 patients were initially included for hybrid IVUS-OCT intracoronary imaging system. After excluding 3 patients with unclear OCT imaging ( Figure 2 ), 74 patients were ultimately included in the study. A total of 82 coronary arteries underwent hybrid IVUS-OCT imaging, including 23 arteries with plaque characteristics analysis and 74 vessels with immediate post-stent evaluations. The left anterior descending artery (LAD) accounted for the highest proportion (54.88%), followed by the right coronary artery (RCA) (26.83%), left circumflex artery (LCX) (14.63%), left main artery (LM) (2.44%), and diagonal branch (D) (1.22%) ( Table 1 ).

**Table 1:** Baseline characteristics and imaging results of the included population.

Characteristic	Analysis population (74 cases)
Basic information	
Age (years), Mean ± SD	61.68 ± 10.61
Male, *n* (%)	53 (71.62%)
Female, *n* (%)	21 (28.38%)
Smoking history, *n* (%)	39 (52.7%)
Drinking History, *n* (%)	21 (28.38%)
BMI (kg/m^2^)	
Missing, *n* (%)	9 (12.16%)
Underweight (<18.5 kg/m^2^), *n* (%)	2 (2.7%)
Normal (18.5–23.9 kg/m^2^), *n* (%)	27 (36.49%)
Overweight (24–27.9 kg/m^2^), *n* (%)	22 (29.73%)
Obesity (≥28 kg/m^2^), *n* (%)	14 (18.92%)
Admission Diagnosis	
Stable Angina, *n* (%)	4 (5.41%)
Unstable Angina, *n* (%)	62 (83.78%)
Non-ST-Segment Elevation Myocardial Infarction, *n* (%)	7 (9.46%)
ST-Segment Elevation Myocardial Infarction, *n* (%)	1 (1.35%)
Medical history	
Hypertension, *n* (%)	58 (78.38%)
Type 2 Diabetes, *n* (%)	33 (44.59%)
Dyslipidemia, *n* (%)	69 (93.24%)
Hyperuricemia, *n* (%)	4 (5.41%)
Chronic Heart Failure, *n* (%)	4 (5.41%)
Old Cerebral Infarction, *n* (%)	14 (18.92%)
Number of interventions	
1, *n* (%)	45 (59.46%)
2, *n* (%)	22 (29.73%)
3, *n* (%)	6 (8.11%)
4, *n* (%)	1 (1.35%)
Lesion type	
Single-Vessel, *n* (%)	11 (16.87%)
Double-vessel, *n* (%)	23 (32.53%)
Triple-vessel, *n* (%)	31 (39.76%)
Left main, *n* (%)	9 (10.84%)
Imaging vessels	
Left Main, *n* (%)	2 (2.44%)
Left Anterior Descending Branch, *n* (%)	45 (54.88%)
Left circumflex Branch, *n* (%)	12 (14.63%)
Right Coronary Artery, *n* (%)	22 (26.83%)
Diagonal Branch, *n* (%)	1 (1.22%)
Stent information	
Number of Stents per Lesion, Mean ± SD `*χ* ± s	1.53 ± 0.69
Length of Stents per Lesion (mm), Mean ± SD:	35.62 ± 19.62
Average Stent Diameter (mm), Mean ± SD	3.03 ± 0.45

### 3.2 Coronary artery plaque characteristics evaluation

**Figure 3:**
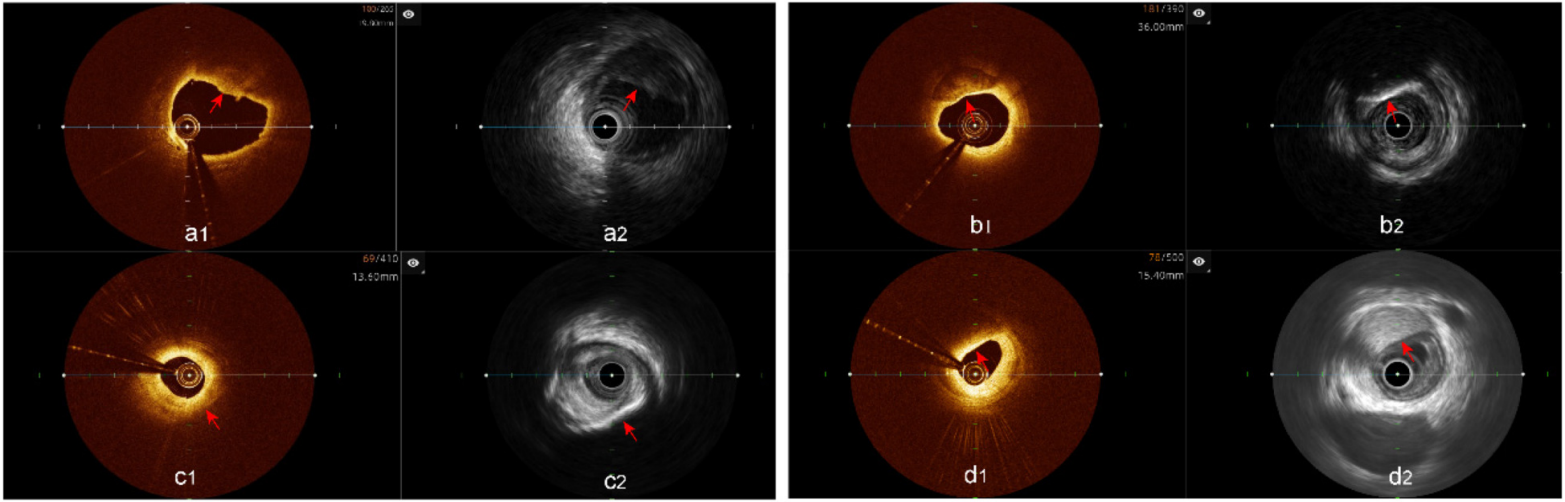
Plaque characterization analysis using hybrid IVUS-OCT imaging system **(a1,a2)** lipid plaques; **(b1,b2)** calcified plaques; **(c1)** OCT showing lipid plaque, **(c2)** IVUS showing calcified plaque; **(d1)** OCT showing lipid plaque, **(d2)** IVUS showing fibrous plaque.

**Figure 4:**
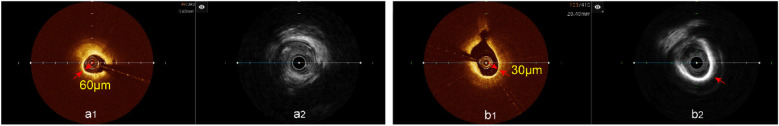
TCFA analysis hybrid IVUS-OCT imaging system. **(a1)** OCT showing TCFA, **(a2)** IVUS showing lipid plaque detected; **(b1)** OCT showing TCFA, **(b2)** IVUS showing calcified plaque.

**Figure 5:**
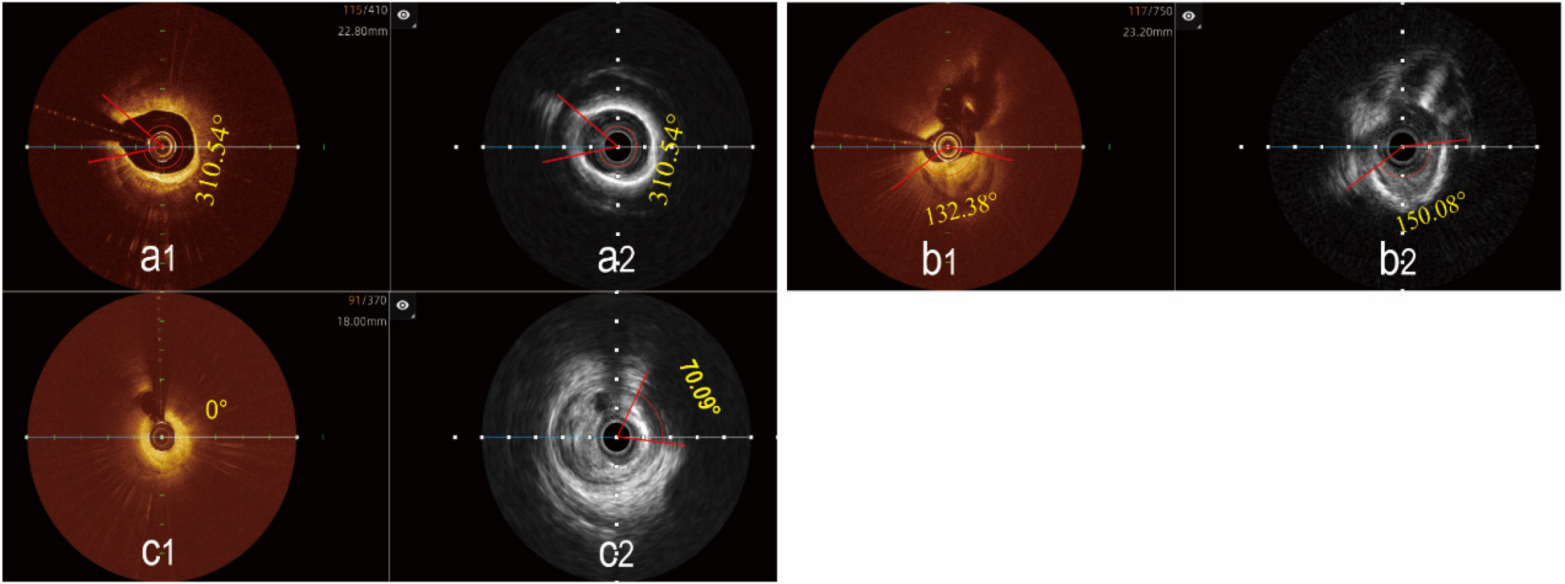
Determination of the maximal calcified plaques arc using the hybrid IVUS-OCT imaging system. **(a1,a2)** The maximal arc of calcification is the same in both images; **(b1)** OCT shows a smaller maximal arc of calcification than IVUS b2; **(c1)** OCT cannot display calcified plaque, **(c2)** IVUS shows the maximal arc of calcification.

**Figure 6:**
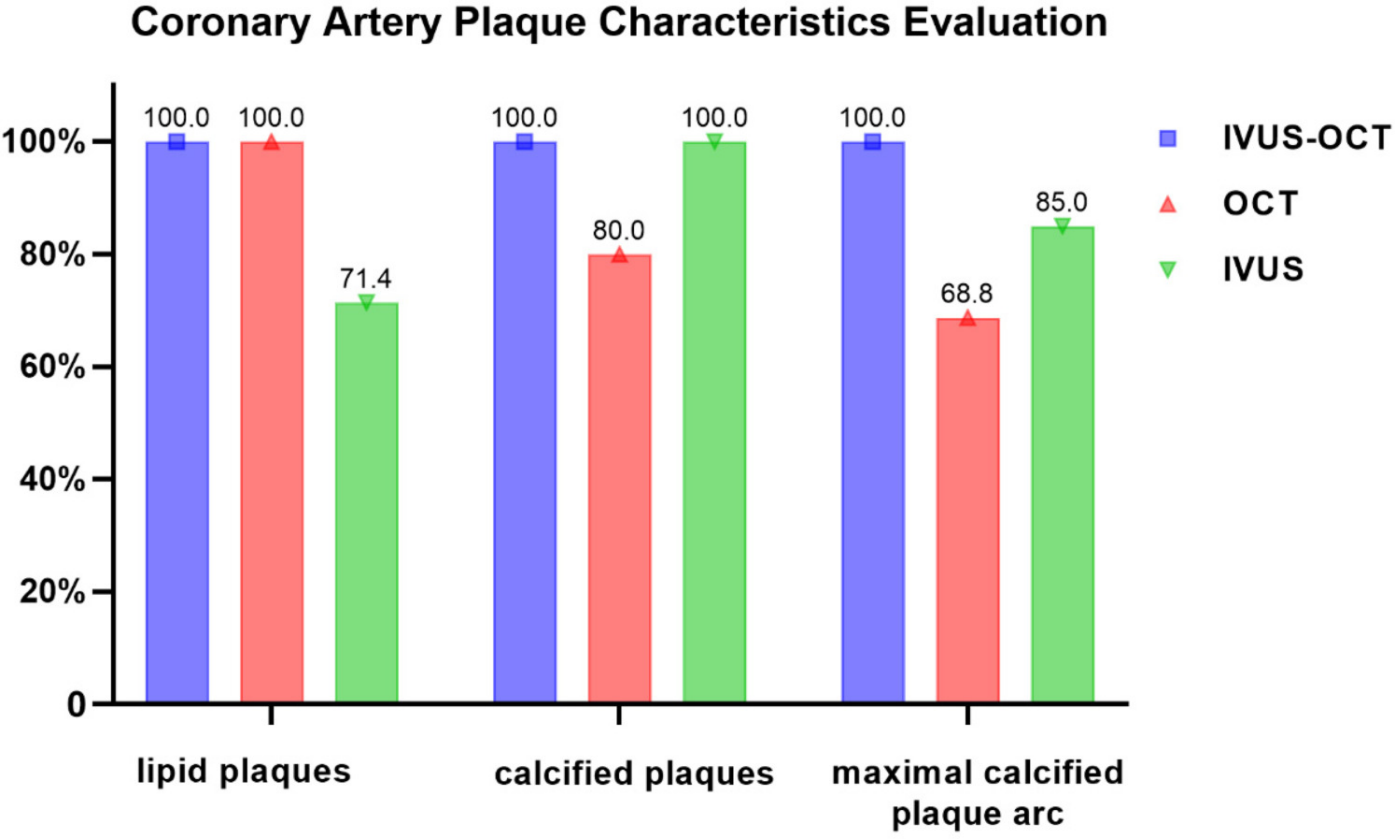
Coronary artery plaque characteristics evaluation.

Analysis of plaque characteristics in 23 hybrid IVUS-OCT pullbacks identified a total of 41 plaques, including 21 lipid plaques, 20 calcified plaques, and 2 of TCFAs (Thin-Cap Fibroatheroma) (Figures 3, 4). OCT alone identified 21 lipid plaques, 16 calcified plaques, including 3 of possible TCFAs, accurately identifying 11 maximal calcified plaque arcs. IVUS alone identified 15 lipid plaques and 20 calcified plaques, accurately identifying 17 maximal calcified plaque arcs (Figure 5). Using the hybrid IVUS-OCT findings as the reference standard, the accuracy of maximal calcified plaque arc identification by OCT was 68.75%. IVUS demonstrated an accuracy of 85% for maximal calcified plaque arc identification but failed to identify any vulnerable plaques (Table 2; Figure 6).

**Table 2:** Coronary artery plaque characteristics evaluation *n* (%).

IVUS-OCT	OCT	IVUS
Lipid Plaques (21)	21 (100)	15 (71.43)
Calcified Plaques (20)	16 (80)	20 (100)
Maximal Calcified Plaque Arc (20)	11 (68.75)	17 (85)
TCFA (2)	3	0 (0)

IVUS, intravascular ultrasound; OCT, optical coherence tomography; TCFA, thin-cap fibroatheroma.

### 3.3 Immediate post-stent evaluation of coronary arteries

Through the Hybrid IVUS-OCT imaging system, 74 coronary arteries were evaluated immediately after stenting, with independent comparisons conducted using both IVUS and OCT.

**Figure 7:**
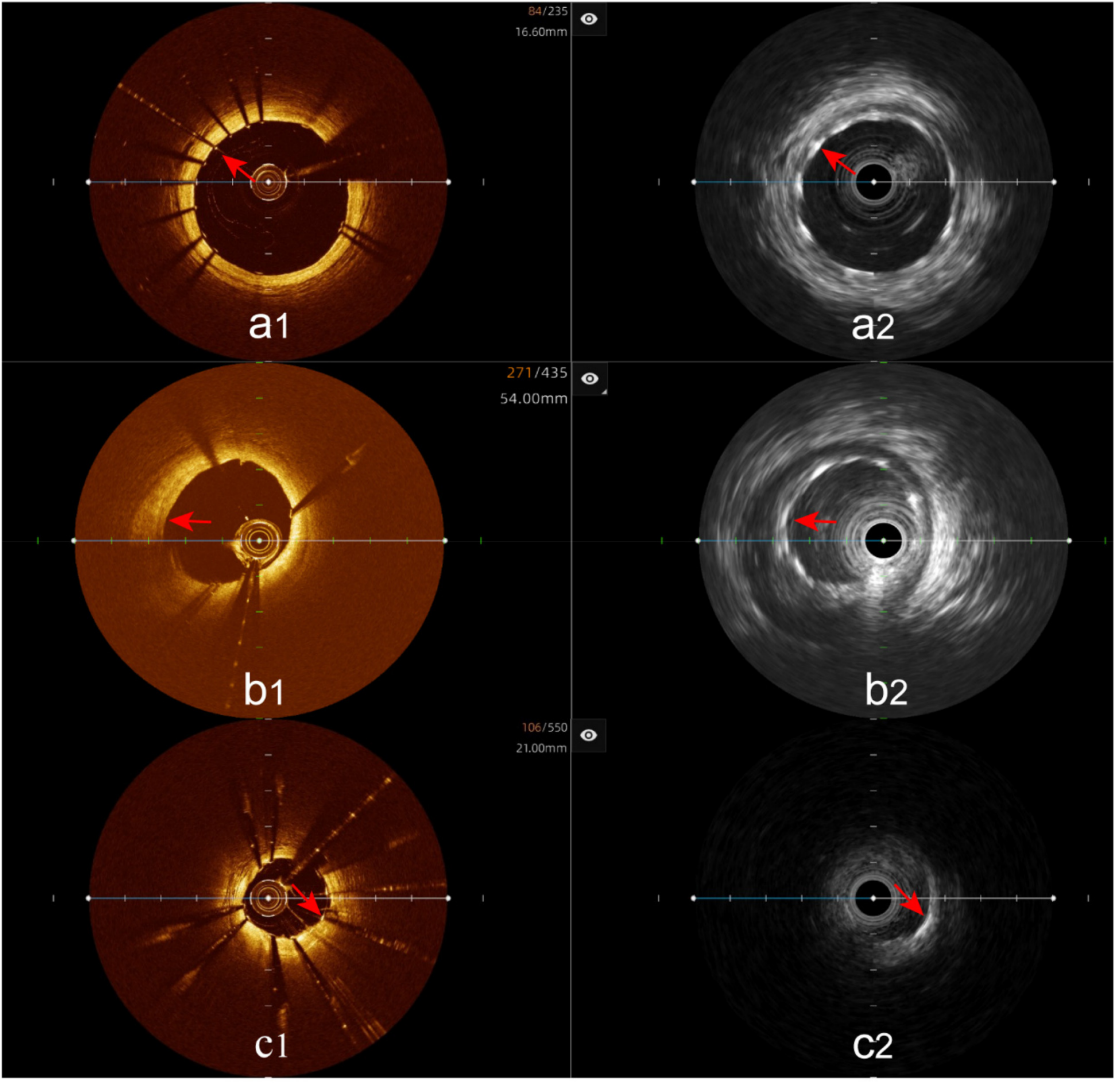
Immediate evaluation of the hybrid IVUS-OCT images after coronary stenting (full clarity of stent capture rate). **(a1,a2)** OCT and IVUS clearly show the stent. **(b1)** OCT shows unclear stent visualization, **(b2)** IVUS clearly shows the stent; **(c1)** OCT clearly shows the stent, **(c2)** IVUS shows unclear stent visualization.

**Figure 8:**
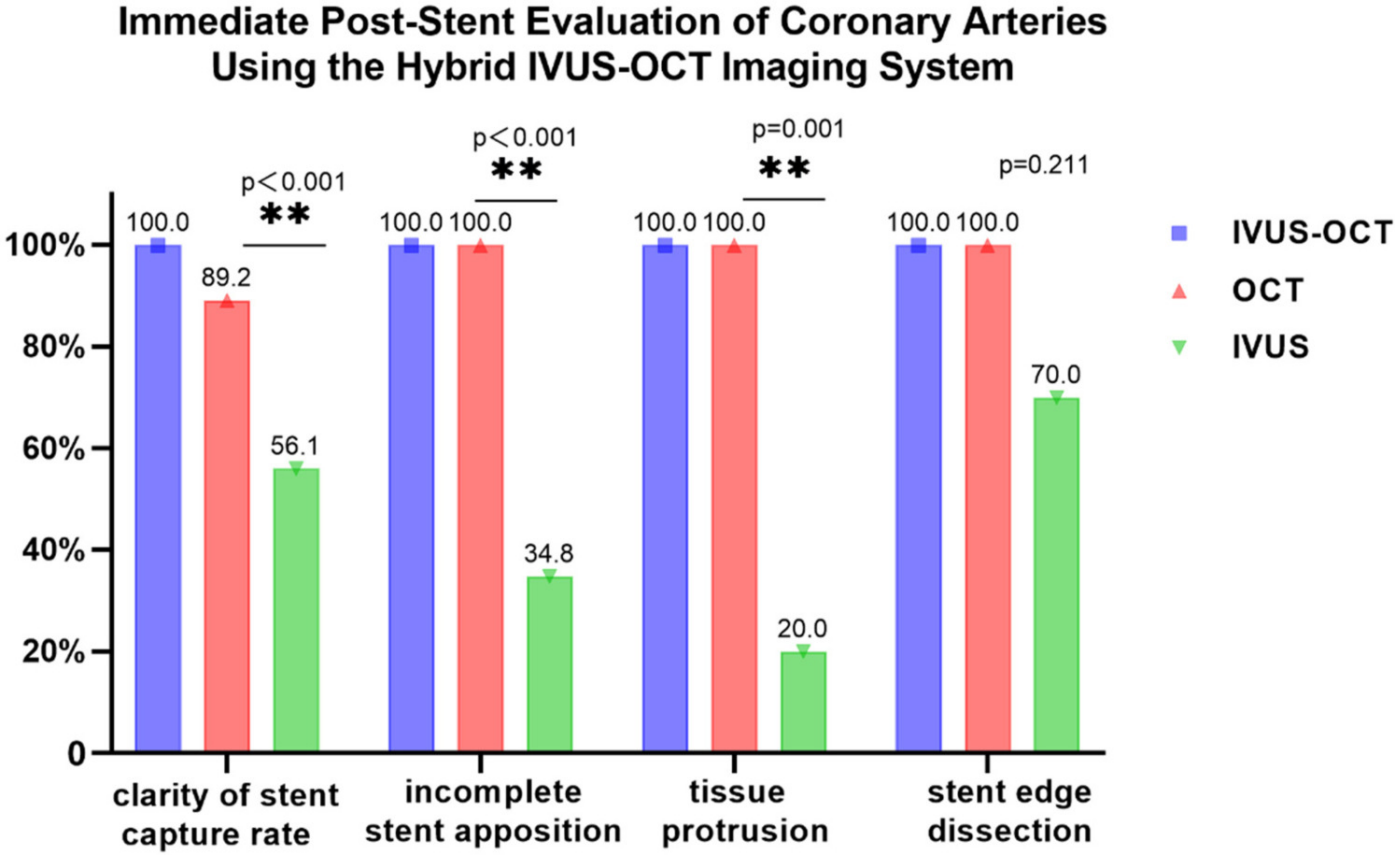
Immediate post-stent evaluation of coronary arteries using the hybrid IVUS-OCT imaging system.

Clear stent capture rate: The hybrid IVUS-OCT images clearly displayed the stents in all lesion vessels, while OCT detecting 66 (89.19%) and IVUS detecting 37 (50%) (*P* < 0.001; Figure 7; Table 3; Figure 8).

**Table 3:** Immediate post-stent evaluation of coronary arteries using the hybrid IVUS-OCT imaging system (*n*, %).

IVUS-OCT	OCT	IVUS	*P*
Full clarity of stent capture rate (74)	66 (89.19)	37 (50)	<0.001
Incomplete stent apposition (23)	23 (100)	8 (34.78)	<0.001
Tissue protrusion (10)	10 (100)	2 (20)	0.001
Stent edge dissection (10)	10 (100)	7 (70)	0.211

IVUS, intravascular ultrasound; OCT, optical coherence tomography.

**Figure 9:**
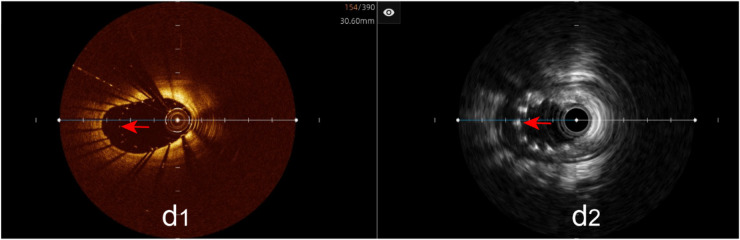
Immediate evaluation of the hybrid IVUS-OCT images after coronary stenting (incomplete stent apposition). **(d1,d2)** OCT and IVUS clearly show incomplete stent apposition.

Incomplete stent apposition: The hybrid IVUS-OCT imaging system identified 23 cases of incomplete stent apposition, compared with OCT detecting 23 (100%) and IVUS detecting 8 (34.78%) (*P* < 0.001; Figure 9; Table 3; Figure 8).

**Figure 10:**
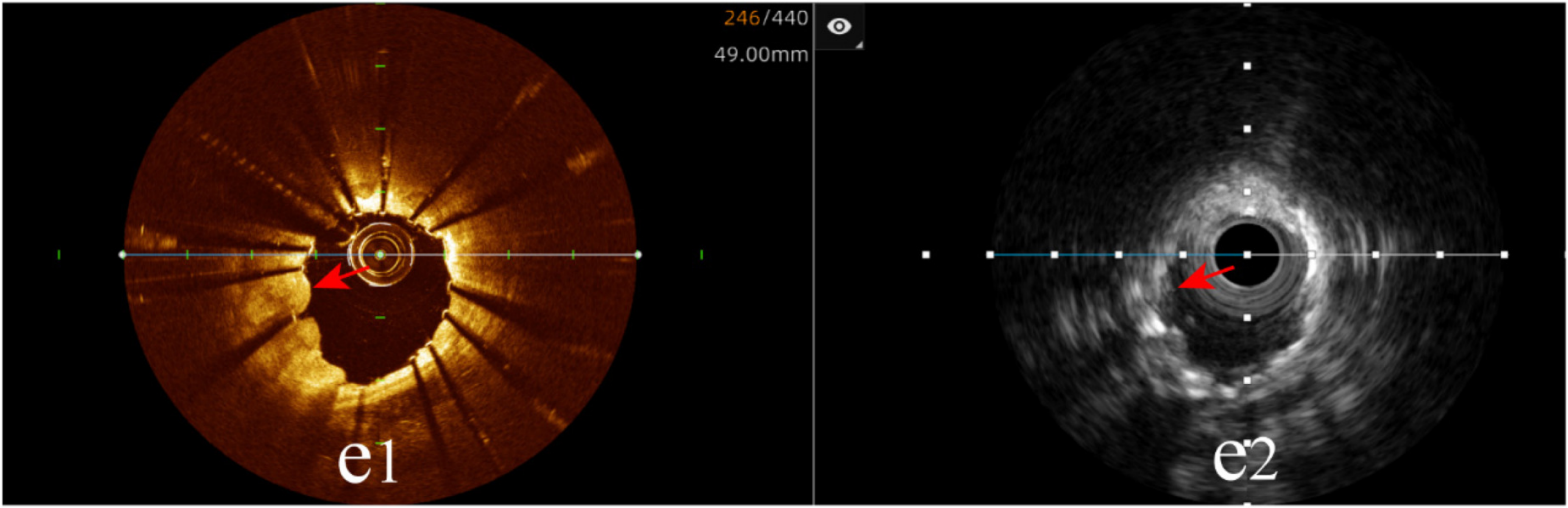
Immediate evaluation of the hybrid IVUS-OCT images after coronary stenting (tissue protrusion). **(e1)** OCT shows tissue protrusion; **(e2)** IVUS cannot visualize tissue protrusion.

Tissue protrusion: The hybrid IVUS-OCT imaging system identified 10 cases of tissue protrusion, compared with OCT detecting 10 (100%) and IVUS detecting 2 (20%) (*P* = 0.001; Figure 10; Table 3; Figure 8).

**Figure 11:**
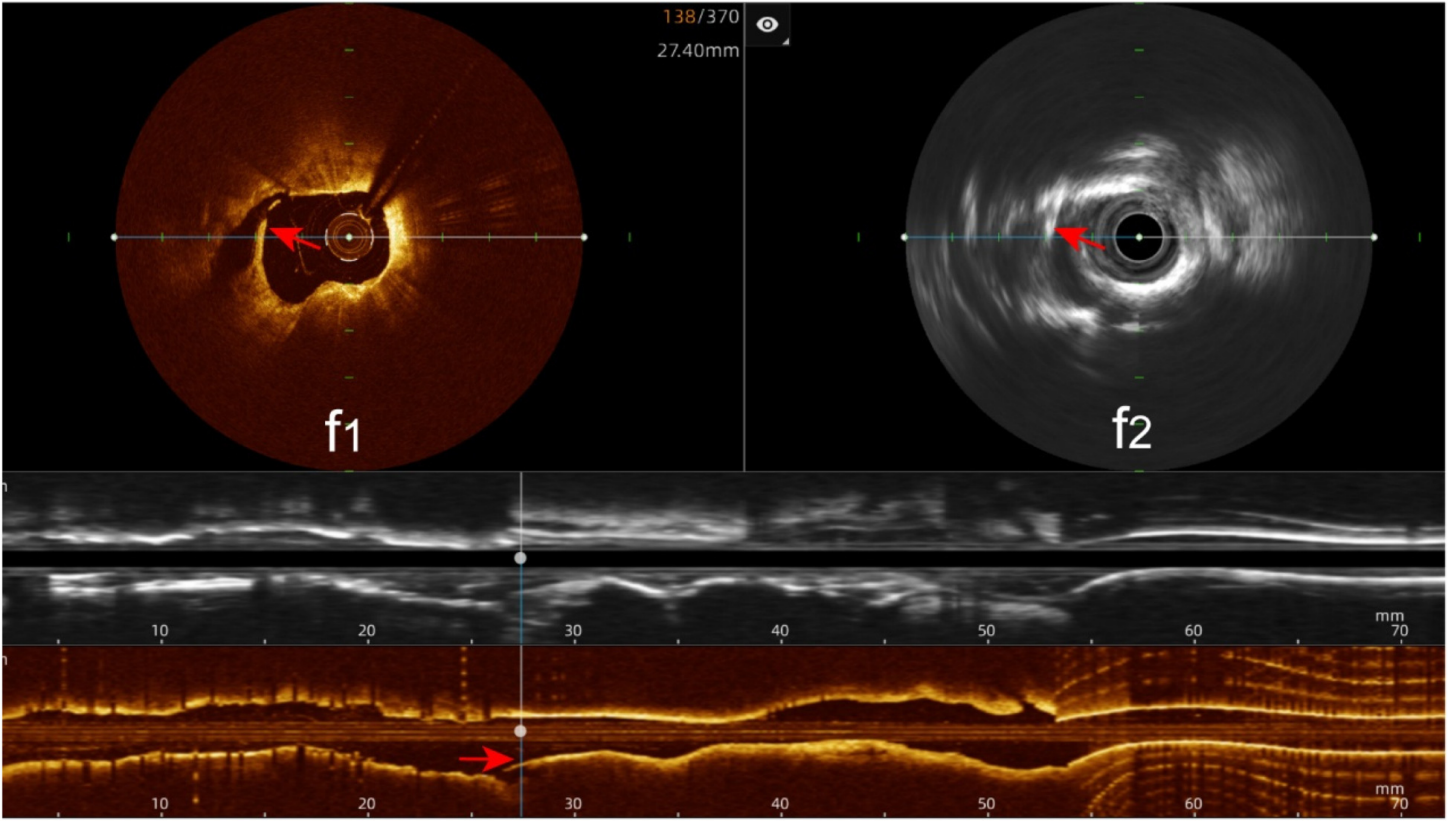
Immediate evaluation of the hybrid IVUS-OCT images after coronary stenting (stent edge dissection). **(f1,f2)** OCT and IVUS clearly show stent edge dissection.

Stent edge dissection: The hybrid IVUS-OCT imaging system identified 10 cases of stent edge dissection, compared with OCT detecting 10 (100%) and IVUS detecting 7 (70%) (*P* = 0.211; Figure 11; Table 3; Figure 8).

## 4 Discussion

The 2024 ESC guidelines' Class Ia recommendation for intravascular imaging (IVI) in complex percutaneous coronary intervention (PCI) underscores its established role in optimizing outcomes (8, 24). However, the inherent limitations of single-modality intravascular ultrasound (IVUS) and optical coherence tomography (OCT)—specifically, IVUS's lower resolution impeding detailed superficial characterization and OCT's limited penetration depth—restrict their capacity for comprehensive lesion assessment (2, 25–27). To bridge this gap, we evaluated a novel hybrid IVUS-OCT intracoronary imaging system designed to synergistically combine these complementary modalities. Our findings demonstrate that this integrated approach significantly outperforms either single-modality IVUS or OCT alone in assessing coronary atherosclerotic plaque characteristics and immediate post-stent outcomes.

In the plaque characteristics analysis, OCT alone identified 21 lipid plaques, 16 calcified plaques, and 3 possible TCFAs, with a maximal calcified plaque arc identification accuracy of 68.75%. IVUS alone identified 15 lipid plaques and 20 calcified plaques, with a maximal calcified plaque arc identification accuracy of 85%. Due to the limited penetration, OCT is unable to visualize deeper plaque components especially beyond lipid plaque or a layer of macrophages. In this study, OCT missed deep calcified plaques and failed to confirm TCFAs due to its inability to characterize plaque components beyond the lipid plaque and macrophages and to measure the size of lipid plaques. There was 3 of the possible TCFA detected by OCT alone, however there was one non-TCFA by hybrid imaging, because there were thin fiber tissue beyond the macrophages. Furthermore, in assessing the maximal calcified plaque arc, OCT is less accurate than IVUS. IVUS defines lipid plaques by visually observing the strength of the plaque echo compared to the echo of the external elastic membrane. The echo strength is influenced not only by tissue density but also by the relative position and arc of the ultrasound catheter to the plaque. Consequently, IVUS can easily confuse lipid plaques with fibrous plaques and may overlook smaller lipid plaques. However, for calcified plaques, IVUS shows good results and can provide accurate calcification information. Moreover, IVUS can evaluate plaque burden and vascular remodeling index, effectively guiding the selection of surgical strategies (13). Although IVUS performs better in identifying calcified plaques compared to OCT, it cannot measure the size of calcified plaques. IVUS also struggles to measure fibrous cap thickness because the lower resolution and cannot assess TCFA. Since plaque characteristics involve numerous parameters, evaluating them comprehensively with a single-modality is challenging (28). This limitation affects the ability to assess the effects of pharmacological interventions on plaques (29), thereby impacting the development of coronary intervention strategies. The hybrid IVUS-OCT imaging system offers dual-modality imaging that compensate for the limitations of each other, allowing for the simultaneous capture of images of plaque characteristics and function, enabling more accurate assessment of plaque characteristics and better guiding clinical decisions.

Regarding immediate post-stent evaluation, our results confirmed that OCT surpassed IVUS in clearly identifying stents, detecting incomplete stent apposition, and identifying tissue protrusion (*P* < 0.05), aligning with previous studies (30). This is attributed to the higher resolution of OCT, enabling it to clearly visualize subtle structural changes surrounding the stent. Nonetheless, OCT and IVUS alone have limitations in immediate post-stent evaluation. The hybrid IVUS-OCT imaging demonstrated excellent performance in this regard, clearly displaying stents and detecting all cases of incomplete stent apposition, tissue protrusion, and stent edge dissection. Clinically, the hybrid IVUS-OCT imaging system provides detailed and accurate immediate post-stent evaluations, helping to avoid the need for repeat procedures and reducing postoperative complications. OCT offers significant advantages for post-stent evaluation, but for patients with suboptimal OCT imaging, IVUS can serve as a complementary analysis tool, reducing repetitive procedures and excessive contrast agent use, thereby decreasing the incidence of cardiovascular adverse events. IVUS also helps to guide stent size selection by providing the measurement of external elastic membrane (EEM) (31–33). The plaque burden can be accurately measured by the hybrid IVUS-OCT imaging system.

The hybrid IVUS-OCT imaging system has been applied clinically recently, and there are few reports regarding its clinical efficacy. While previous studies have compared the clinical effects of OCT and IVUS separately, they did not scan images simultaneously, which means they could not ensure that OCT and IVUS images were from the same frame. In this study, both plaque characteristic analysis and post-stent evaluation showed that the hybrid IVUS-OCT imaging system performed well in clinical guidance, realizing the benefits of complementary imaging (34). It enables preoperative strategy selection or postoperative outcome evaluation leveraging the other modality if one underperforms, thereby minimizing additional procedures and excessive contrast agent administration. Additionally, it alleviates the dilemma of selecting the appropriate coronary intravascular imaging modality, especially during the interventional treatment of complex coronary lesions (35). It is important to note that since OCT imaging requires contrast agent flushing, careful consideration is necessary for patients with left main lesions, severe heart failure, or renal dysfunction (36). In these cases, the hybrid system can be operated in IVUS mode without requiring contrast agent flushing.

In this study, no adverse reactions related to coronary intravascular imaging were observed in any patients. Several limitations of this study warrant consideration. First, the single-center design and relatively small sample size limit the generalizability of the findings. Second, the evaluation of plaque characteristics and post-stent outcomes relied solely on intravascular imaging modalities lacking histological gold standard validation.Third, the lack of long-term clinical follow-up data precludes assessment of whether the superior lesion characterization and stent assessment provided by the hybrid system translates to improved clinical outcomes (e.g., reduced major adverse cardiovascular events, target lesion revascularization) compared to single-modality guidance. Finally, while image analysis was performed offline by two independent researchers with adjudication by a third in case of disagreement, inherent subjectivity in interpreting intravascular images, especially for subtle findings, remains a potential source of bias.

This study demonstrates the advantages of the hybrid IVUS-OCT intravascular imaging system and its excellent clinical guidance potential. Future studies should be conducted with larger sample sizes, multi-center involvement, and randomized designs to obtain more valuable clinical data on the clinical application of the hybrid IVUS-OCT imaging system.

## Data Availability

The raw data supporting the conclusions of this article will be made available by the authors, without undue reservation.
